# *Vibrio natriegens* as Host for Expression of Multisubunit Membrane Protein Complexes

**DOI:** 10.3389/fmicb.2018.02537

**Published:** 2018-10-25

**Authors:** Lena Schleicher, Valentin Muras, Björn Claussen, Jens Pfannstiel, Bastian Blombach, Pavel Dibrov, Günter Fritz, Julia Steuber

**Affiliations:** ^1^Institute of Microbiology, University of Hohenheim, Stuttgart, Germany; ^2^Mass Spectrometry Core Facility, University of Hohenheim, Stuttgart, Germany; ^3^Institute of Biochemical Engineering, University of Stuttgart, Stuttgart, Germany; ^4^Department of Microbiology, University of Manitoba, Winnipeg, MB, Canada; ^5^Institute for Neuropathology, University of Freiburg, Freiburg im Breisgau, Germany

**Keywords:** *Vibrio natriegens*, membrane proteins, multisubunit complexes, expression, Na^+^-translocating NADH:quinone oxidoreductase, multiple resistance and pH related antiporter, Strep-Tag

## Abstract

*Escherichia coli* is a convenient host for the expression of proteins, but the heterologous production of large membrane protein complexes often is hampered by the lack of specific accessory genes required for membrane insertion or cofactor assembly. In this study we introduce the non-pathogenic and fast-growing *Vibrio natriegens* as a suitable expression host for membrane-bound proteins from *Vibrio cholerae*. We achieved production of the primary Na^+^ pump, the NADH:quinone oxidoreductase (NQR), from *V. cholerae* in an active state, as indicated by increased overall NADH:quinone oxidoreduction activity of membranes from the transformed *V. natriegens*, and the sensitivity toward Ag^+^, a specific inhibitor of the NQR. Complete assembly of *V. cholerae* NQR expressed in *V. natriegens* was demonstrated by BN PAGE followed by activity staining. The secondary transport system Mrp from *V. cholerae*, another membrane-bound multisubunit complex, was also produced in *V. natriegens* in a functional state, as demonstrated by *in vivo* Li^+^ transport. *V. natriegens* is a promising expression host for the production of membrane protein complexes from Gram-negative pathogens.

## Introduction

The heterologous expression and purification of proteins allows their biochemical characterization, use in industrial processes, and development of commercial goods (Rosano and Ceccarelli, 2014). The traditional microbial system for heterologous expression of proteins is *Escherichia coli* with high levels of gene expression and scalability of experiments. However, the production of recombinant proteins, especially membrane-bound proteins, represents the bottleneck of many biotechnological processes. Here we introduce *Vibrio natriegens* as a new host for heterologous expression of multisubunit membrane proteins in their active state.

*Vibrio natriegens* is a Gram-negative, marine, halophilic, non-pathogenic bacterium from the family *Vibrionaceae*. Although it was first described more than 50 years ago (Payne, 1958), only a few studies of *V. natriegens* exist so far. *V. natriegens* has the fastest growth rate of any known non-pathogenic organism with a doubling time of <10 min at optimal growth conditions (Payne, 1958; Eagon, 1962; Weinstock et al., 2016; Hoffart et al., 2017); in comparison, *E. coli* has a doubling time of 20 min. For this enormous growth rate an extremely high rate of protein synthesis is required, which is due to increased amounts of ribosomes (Aiyar et al., 2002). In addition, genetic engineering tools were successfully developed for the manipulation of cellular metabolism and the heterologous expression of proteins in *V. natriegens* (Weinstock et al., 2016; Hoffart et al., 2017). Recent studies reported a *V. natriegens-*based cell-free protein synthesis system (CFPS) for synthesis of the model protein eGFP (Failmezger et al., 2018). *V. natriegens* fulfills basic requirements for biotechnological applications (Hoffart et al., 2017): bioreactor cultivations of *V. natriegens* in minimal medium with glucose resulted in a remarkable high *qs* (specific consumption rate), which was at least two times higher than those of *E. coli*, *Bacillus subtilis*, *Corynebacterium glutamicum* and yeast, the traditional microbial systems. Consequently, *V. natriegens* is a very interesting organism, which could become a tool for different fields of biotechnological research and development.

The heterologous expression of membrane protein complexes in their active state is especially difficult since subunits must integrate into the host’s membrane, and must assemble in a correct manner. Both processes have to be coordinated to obtain a fully functional membrane protein complex. Here we examine the potential of *V. natriegens* as a new expression host for membrane proteins from *Vibrio cholerae*, namely the primary transport system NQR and the secondary transport system Mrp. The NQR is a respiratory enzyme which is widespread among pathogenic bacteria (Steuber et al., 2014a,b). It is composed of six subunits (NqrABCDEF) and contains several cofactors: one FAD, two iron-sulfur centers, one riboflavin, and two covalently bound flavin mononucleotides (FMN). Subunit NqrF harbors FAD and catalyzes the initial oxidation of NADH which can be specifically inhibited by silver ions (Steuber et al., 1997). The membrane-bound NqrB and C subunits each carry one covalently linked FMN which can be visualized by fluorography (Vorburger et al., 2016). Mrp is widely distributed among bacteria and archaea, especially among the organisms that need to adapt to elevated salt and/or high pH environments (Swartz et al., 2005; Dzioba-Winogrodzki et al., 2009). The Mrp from *V. cholerae* contains six subunits and catalyzes the electrogenic efflux of cytoplasmic Na^+^, Li^+^, and K^+^ ions outward in a coupled reaction that transports external H^+^ inward (Swartz et al., 2005; Dzioba-Winogrodzki et al., 2009). It is suggested, that the Mrp is needed for maximum protection of *V. cholerae* cells against alkali cations in neutral and alkaline environments. Furthermore, Mrp protects the Na^+^ sensitive *E. coli* EP432 from high concentrations of toxic Li^+^ (Dzioba-Winogrodzki et al., 2009).

In this study, we demonstrate that *V. natriegens* is a suitable expression host for functional membrane proteins. It is also an attractive non-pathogenic *Vibrio* strain for studying functions of proteins from pathogenic *Vibrio* species.

## Results

### Detection of Subunit NqrA of NQR-ST Complex Expressed in *V. natriegens* by Immune Staining After Western Blotting

**FIGURE 1:**
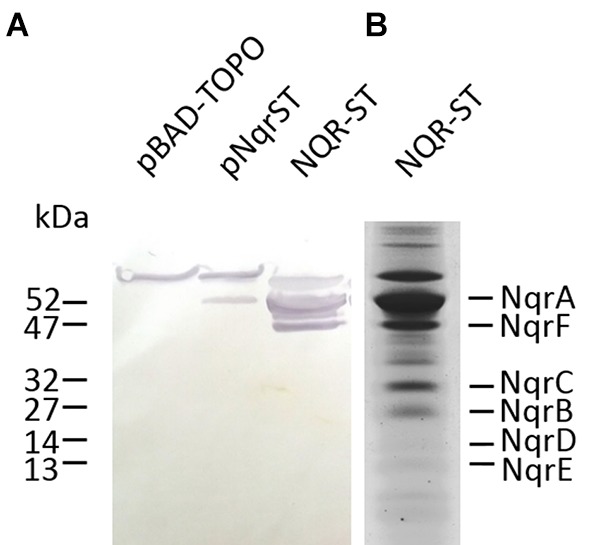
Detection of NQR by immunostaining of Strep-NqrA. After protein expression, the membranes from *Vibrio natriegens* pNqrST were isolated and loaded onto SDS-PAGE. **(A)** Proteins were transferred to a nitrocellulose membrane and NqrA fused to the Strep-tag was detected by a chromogenic reaction. Lane 1, membranes from *V. natriegens* pBAD-TOPO (100 μg); lane 2, membranes from *V. natriegens* pNqrST (100 μg); lane 3, positive control (purified NQR-ST complex, 50 μg). **(B)** Detection of six Nqr subunits in NQR-ST after separation on a 10% SDS gel with 6 M urea. Hydrophobic NqrD and NqrE stain poorly in Coomassie.

So far, expression of NQR from *V. cholerae* was only reported in its endogenous host. Using an expression vector conferring an N-terminal Strep-Tag to the cytoplasmic NqrA subunit, production of *V. cholerae* NQR produced in *V. natriegens* was confirmed with western blotting followed by immune detection (Figure 1A). Strep-tagged NqrA with an apparent molecular mass of 53.5 kDa was detected in membranes from *V. natriegens* cells transformed with pNqrST and with purified NQR-ST complex, but not in membranes from *V. natriegens* cells transformed with the control vector, pBAD-TOPO. In all three samples, we observed an additional immunogenic protein with an apparent mass of 58 kDa, which probably represents a biotin-containing protein of the *Vibrio* expression hosts (Halang et al., 2013). Note that subunit NqrA is prone to proteolytic degradation (Vohl et al., 2014), resulting in an additional band below NqrA which was detected by immunostaining of the purified NQR-ST complex.

As previously reported for the NQR complex carrying a His-tag at the N-terminus of subunit NqrA (Tao et al., 2008; Steuber et al., 2014b), the purified NQR-ST complex with additional Strep-Tag contained all six expected subunits, as confirmed by SDS-PAGE (Figure 1B). To separate the hydrophobic NqrB and NqrC subunits the SDS-PAGE was performed in the presence of 6 M urea (Hayashi et al., 2001). Note that the small, hydrophobic NqrD and NqrE subunits stain only weakly with Coomassie.

### Detection of Subunits NqrB and NqrC of NQR-ST Complex Expressed in *V. natriegens* by *in gel* Fluography

**FIGURE 2:**
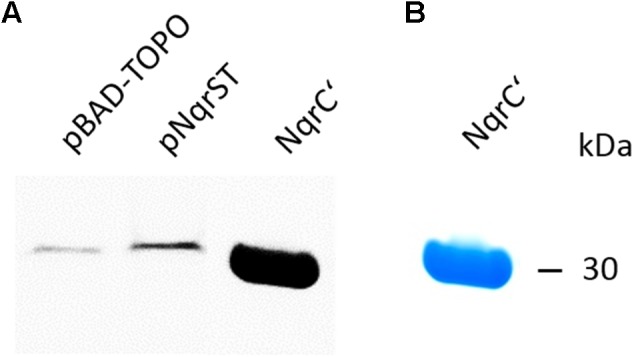
Detection of the membrane-bound NqrB and NqrC subunits of NQR in *V. natriegens* membranes by in gel fluorography. Membranes from *V. natriegens* were separated on SDS-PAGE. **(A)** Lane 1, membranes from *V. natriegens* pBAD-TOPO (100 μg); lane 2, membranes from *V. natriegens* pNqrST (100 μg); lane 3, positive control (NqrC’, 40 μg). **(B)** SDS gel of the positive control (NqrC’) stained with Coomassie.

The subunits NqrB and NqrC exhibit fluorescence due to their covalently bound flavins (Hayashi et al., 2001). After separation of membranes from *V. natriegens* cells transformed with pNqrST or the control vector on SDS-PAGE without urea, we observed a single fluorescent band in both cases (Figure 2A). Subunits NqrB and NqrC co-migrate under these conditions. Fluorescence intensity was increased in the NQR-ST membranes when compared to membranes from control membranes, in accord with the expected overproduction. The weaker signal in control membranes resulted from the endogenous NqrB (accession number UNIPROT: A0A1B1EED7) and NqrC (accession number UNIPROT: A0A1B1EEE9) subunits from the NQR coded on region 455785–461628 of chromosome 1 from *V. natriegens*. These results were also confirmed by mass spectrometry analysis (Table 1 and Supplementary Table S1). As a positive control exhibiting flavin fluorescence, the purified NqrC’ subunit was used comprising the covalently attached FMN but lacking the N-terminal transmembrane helix (Vohl et al., 2014). Accordingly, the protein runs at a lower apparent mass in SDS-PAGE compared to native NqrC. The purity of the control NqrC’ was demonstrated by SDS-PAGE and Coomassie staining (Figure 2B).

**Table 1:** Identification of Nqr subunits in *V. natriegens* pBAD-TOPO and *Vibrio natriegens* pNqr-ST by mass spectrometry.

Number of identified peptides				NqrA	NqrB	NqrC	NqrD	NqrE	NqrF
Fluorescent proteins in SDS-PAGE of membranes	*V. natriegens* pBAD-TOPO				3	12			
	*V. natriegens* pNqr-ST				2	14			
NADH dehydrogenases in BN PAGE	*V. natriegens* pBAD-TOPO	Holo-complex Box 2		8	2	4	2	1	3
		Sub-complex	Box 4	3		3			
			Box 6	2		4			2
	*V. natriegens* pNqr-ST	Holo-complex Box 1		13	2	13	1		11
		Sub-complex	Box 3	1		2			
			Box 5	3					3

*To confirm the presence of NqrB and NqrC, fluorescent protein bands from membranes separated by SDS-PAGE were analyzed (Figure 2A). To describe the composition of NQR complexes, bands with NADH dehydrogenase activity after BN PAGE (indicated by boxes in Figure 5) were analyzed. The number of Nqr-derived peptides identified in each sample varies from 1 to 14. For peptide sequences see Supplementary Table S1.*

### Ag^+^ Inhibits NADH:Quinone Oxidoreduction Activity of *V. natriegens* pNqrST Membranes

**FIGURE 3:**
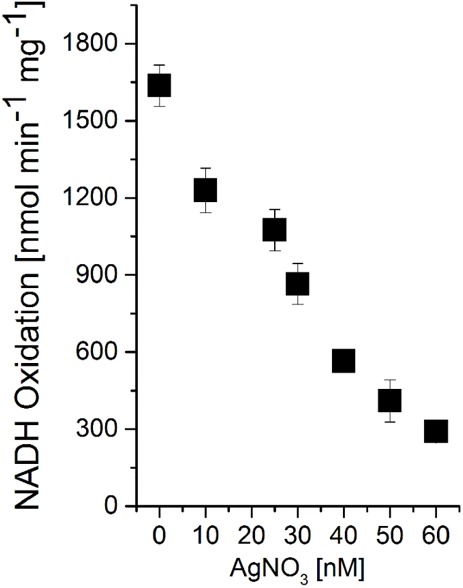
Strong inhibition of NADH:quinone oxidoreduction activity of *V. natriegens* pNqrST membranes by Ag^+^. Membranes were incubated with varying amounts of AgNO_3_ for 5 min at RT. The final Ag^+^ concentration in the assay is indicated. Average and standard deviation from three biological replicates are shown.

**FIGURE 4:**
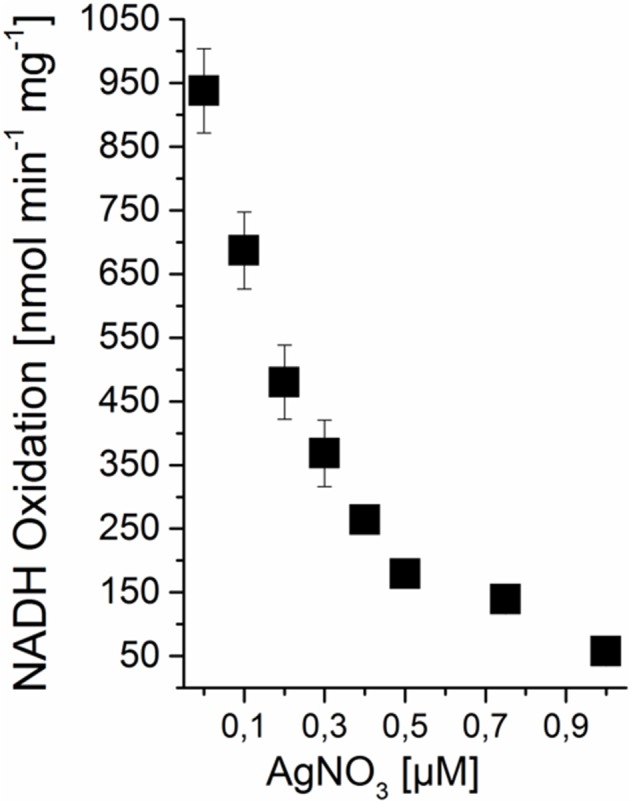
Unspecific Ag^+^ inhibition of NADH:quinone oxidoreduction activity of *V. natriegens* pBAD-TOPO membranes. Membranes were incubated with varying amounts of AgNO_3_ for 5 min at RT. The final Ag^+^ concentration in the assay is indicated. Average and standard deviation from three biological replicates are shown.

The inhibition of the NQR from *V. cholerae* with nanomolar concentrations of Ag^+^ is a typical property of this enzyme (Steuber et al., 1997). Silver ions specifically and irreversibly inactivate the NQR by binding to the NADH-oxidizing NqrF subunit of the complex. With membranes from *V. natriegens* pNqrST, the oxidation of NADH with varying amounts of AgNO_3_ was examined (Figure 3). Without inhibitor, a specific activity of ∼1600 nmol min^-1^ mg^-1^ was observed, which decreased with increasing concentrations of silver nitrate. Ag^+^ in nanomolar concentration range (20–40 nM AgNO_3_) led to a half-maximal inactivation of NADH oxidation activity. The membranes from *V. natriegens* pBAD-TOPO also showed NADH oxidation activity, due to the endogenous NQR (Figure 4). Nevertheless, a significant lower activity with ∼950 nmol min^-1^ mg^-1^ was observed, compared to *V. natriegens* pNqrST. The NADH oxidation activity of membranes from *V. natriegens* pBAD-TOPO could also be inhibited with Ag^+^, but much higher concentrations of the inhibitor were needed, with half-maximal inactivation in the range of 0.2–0.3 μM AgNO_3_.

### Solubilized *V. natriegens* pNqrST Membranes Contain a Protein Complex With NADH Dehydrogenase Activity

**FIGURE 5:**
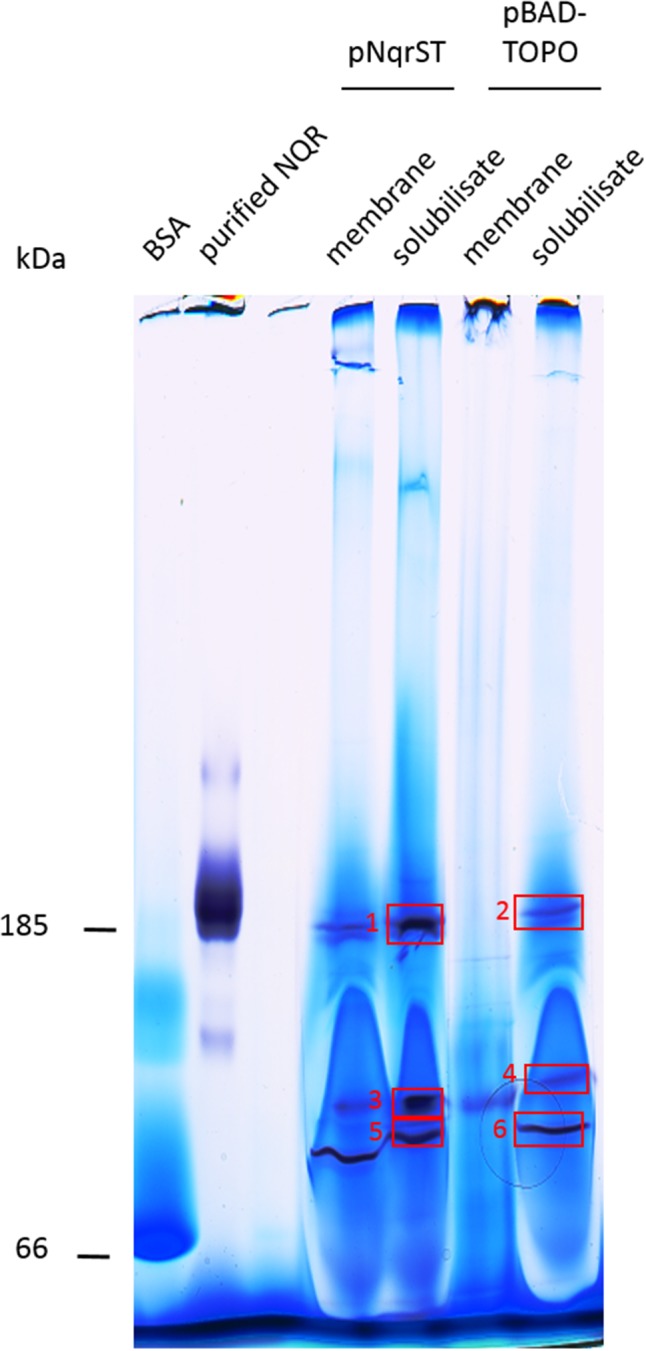
Blue native PAGE of *V. natriegens* membranes and detection of NADH dehydrogenase activity. Membranes and membranes solubilized with 1% DDM were separated by BN PAGE with a gradient from 4 to 16% acrylamide in the gel (100 μg solubilized membrane, or 200 μg membrane, per lane). Protein bands, which contained NADH dehydrogenase complexes or sub-complexes thereof, were identified by *in gel* activity stain. Boxes indicate proteins subjected to mass spectrometry analysis. Upper band represents holo-NQR, the lower bands a sub-complex of NqrA, NqrF, and NqrC (Table 1 and Supplementary Table S1). Negative control, BSA (50 μg). Positive control, purified His-tagged NQR (4 μg).

To further characterize the NADH dehydrogenase activity, solubilized membrane proteins were separated in a Blue Native PAGE (BN PAGE) followed by *in gel* activity staining to detect proteins with NADH dehydrogenase activity. Figure 5 shows the results for the membranes and solubilized membrane proteins of *V. natriegens* pNqrST and *V. natriegens* pBAD-TOPO, respectively. With the solubilized membranes, a band around 185 kDa molecular weight was stained due to precipitation of nitro blue diformazan (Figure 5, boxes 1, 2), indicating NADH dehydrogenase activity. These bands showed the same apparent mass as the purified NQR complex exhibiting *in gel* activity which was used as control. Staining of the *V. natriegens* pBAD-TOPO band at 185 kDa (Figure 5, box 2) was less intensive than with solubilized pNqrST membranes (Figure 5, box 1), indicating that endogenous NQR from *V. natriegens* was also solubilized with 1% DDM and exhibited a similar migration behavior as the Strep-His-tagged NQR from *V. cholerae*. Mass spectrometry analysis confirmed the presence of all six Nqr subunits in these protein bands (Table 1 and Supplementary Table S1). We conclude that holo-NQR complex from *V. cholerae* was produced in *V. natriegens*. Furthermore, two sharp bands migrated roughly between the NQR complex (185 kDa) and BSA (66 kDa) in each solubilization sample (Figure 5, box 3/box 5, box 4/box 6), indicating the presence of smaller NQR sub-complexes. Mass spectrometry analysis identified NqrA and NqrC in both upper protein bands (Figure 5, boxes 3, 4). NqrA, NqrC and NqrF were identified in the lower protein band of solubilized *V. natriegens* pBAD-TOPO membranes (Figure 5, box 6). NqrA and NqrF were identified in the lower protein band of solubilized *V. natriegens* pNqrST membranes (Figure 5, box 5, Table 1 and Supplementary Table S1). The NADH oxidizing NqrF subunit could not be detected in the upper protein bands (Figure 5, boxes 3, 4) by mass spectrometry, probably because of limited sensitivity. However, its presence is very likely since neither NqrA nor NqrC exhibit NADH dehydrogenase activity.

### Mrp Is Expressed in *V. natriegens*

**FIGURE 6:**
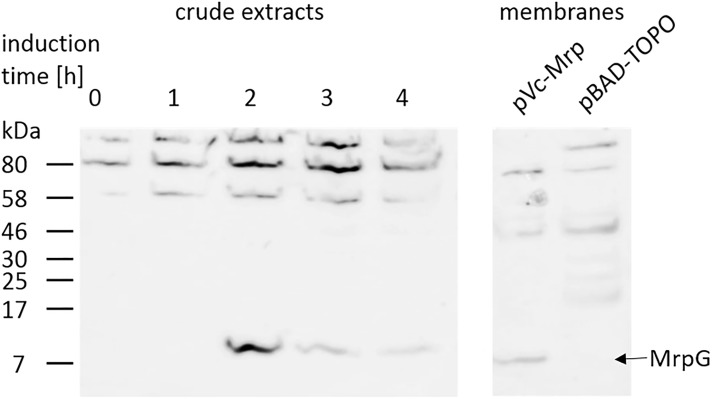
Detection of V5-epitope from MrpG. Expression of pVc-Mrp was induced with 0.02% (w/v) L-arabinose and cells were harvested after indicated times (0–4 h). Cell pellets from 1 ml cell suspension with OD_600_ = 1 or membranes from *V. natriegens* pVc-Mrp (100 μg) or *V. natriegens* pBAD-TOPO (100 μg) were separated on SDS-PAGE and proteins were transferred to a nitrocellulose membrane. MrpG (fused to a V5-epitope) was detected by immunostaining (black arrow).

Like NQR, the *mrp* operon from *V. cholerae* encoded by the pVc-Mrp plasmid had not been expressed in *V. natriegens* before, so we first performed expression tests with different L-arabinose concentrations (0.2, 0.02, and 0.002%) and various induction times. Cells broken by treatment with SDS sample buffer were analyzed by SDS-PAGE. MrpG was detected by western blotting and immune detection with anti-V5-HRP antibodies and chemiluminescence (apparent molecular mass of MrpG with His_6_ tag and V5-epitope tag: 13.5 kDa) (Figure 6). The strongest signal was observed with 0.02% L-arabinose after 2 h of induction. Up to 2 h induction, no MrpG-V5 was detected. After 2 h induction time, the expression levels declined continuously. Furthermore, three protein bands with a higher molecular weight became visible in every crude extract sample, even in the negative control where no L-arabinose was added. These proteins were already visible after 30 s of exposure (data not shown), whereas the MrpG-V5 protein band required 2 min exposure time. Membranes from *V. natriegens* pVc-Mrp and *V. natriegens* pBAD-TOPO were also analyzed in a western blot. Only the *V. natriegens* pVc-Mrp membranes showed the expected band at ∼13 kDa, indicating the presence of MrpG-V5 in *V. natriegens* membranes.

### Functional Mrp Permits Growth of *V. natriegens* at High Li^+^ Concentrations

**FIGURE 7:**
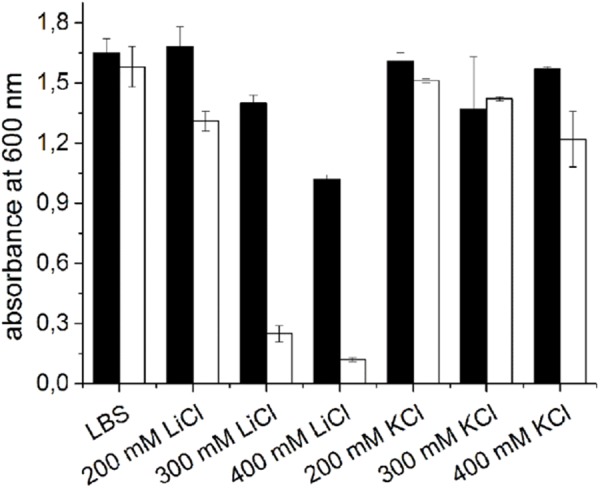
Li^+^ inhibition of growth of *V. natriegens* in the absence or presence of *V. cholerae* Mrp complex. Cells were transformed with either the pVc-Mrp (black bars) or with the pBAD-TOPO plasmid (white bars) and grown aerobically in 10 ml LBS in the presence of 0.2% L-arabinose and varying LiCl or KCl concentrations. After 20 h, the OD_600_ was determined. Averages and standard deviations from three biological replicates are shown.

Dzioba-Winogrodzki et al. (2009) postulated that the Mrp from *V. cholerae* is a cation/H^+^ antiporter, protecting the Na^+^ sensitive strain *E. coli* EP432 from stress caused by high external concentrations of Li^+^. To analyze if Mrp also protects *V. natriegens* from high Li^+^ concentrations, growth experiments in glass test tubes were performed. First, the critical Li^+^ concentration preventing growth of the *V. natriegens* strains was determined (Figure 7). *V. natriegens* pVc-Mrp and *V. natriegens* pBAD-TOPO grown in LBS medium showed no significant differences in the optical densities after 20 h of incubation without added Li^+^. In presence of 300 mM or 400 mM LiCl, *V. natriegens* pVc-Mrp showed a much higher optical density compared to *V. natriegens* pBAD-TOPO after 20 h. With 300 mM LiCl added, *V. natriegens* pVc-Mrp reached a final OD_600_ of 1.4 ± 0.04, whereas *V. natriegens* pBAD-TOPO had a final OD_600_ of 0.25 ± 0.04. With 400 mM LiCl, *V. natriegens* pVc-Mrp reached a final OD_600_ of 1.02 ± 0.02 and *V. natriegens* pBAD-TOPO had a final OD_600_ of 0.12 ± 0.01. We conclude that Mrp permits the growth of *V. natriegens* at high Li^+^ concentrations, which inhibits the growth of *V. natriegens* pBAD-TOPO. Cells grown in presence of different KCl concentrations were used as a control, revealing no toxic effect of K^+^. Therefore, the observed inhibition of *V. natriegens* pBAD-TOPO by LiCl was not caused by elevated chloride concentrations.

**FIGURE 8:**
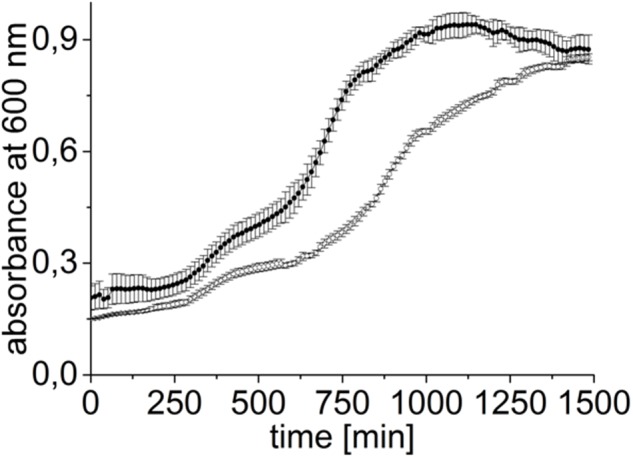
Mrp expression confers a growth advantage to *V. natriegens* at high [Li^+^]. Cells were transformed with either the pVc-Mrp (closed circles) or with the pBAD-TOPO plasmid (empty circles) and grown aerobically in LBS in the presence of 0.2% L-arabinose and 400 mM LiCl at 37°C in a 96-well plate. Averages and standard deviations from three biological replicates are shown.

We also monitored growth of *V. natriegens* pVc-Mrp in presence of 400 mM LiCl over time with a plate reader (Figure 8). Growth of the *V. natriegens* pBAD-TOPO reference strain was followed in parallel. Again, the strains exhibit different growth phenotypes. Although both strains reached nearly the same final OD_600_ of 0.85 ± 0.13 (pBAD-TOPO) and 0.87 ± 0.04 (pVc-Mrp), *V. natriegens* pVc-Mrp reached its highest cell density of 0.9 ± 0.03 already after 17 h, compared to 24 h with *V. natriegens* pBAD-TOPO (Figure 8). Aeration of cultures is improved in microtiter plates compared to glass tubes, and we conclude that the *V. cholerae* Mrp in its active state protected *V. natriegens* from toxic LiCl under both conditions.

## Discussion

The expression of multisubunit membrane proteins faces two major problems. First, the coordinated expression of the subunits and second, the correct assembly and insertion of the complex into the membrane. Membrane protein complexes normally cannot be reconstituted by expression of the subcomplexes only. They need, e.g., interaction partners for stable folding and function (Zorman et al., 2015). Until now, this problem was solved mainly with co-expression of multiple genes using, e.g., the Duet (Novagen) vectors (Tolia and Joshua-Tor, 2006). These vectors have multiple cloning sites, five origins of replication and four antibiotic selection markers, allowing the simultaneous expression of up to eight proteins (Tolia and Joshua-Tor, 2006). The MultiBac system is another tool (Pelosse et al., 2017). It is a baculovirus/insect expression system specifically engineered for production of functional multiprotein complexes (Berger et al., 2004; Fitzgerald et al., 2006; Pelosse et al., 2017). In this study, we were able to express two membrane-bound protein complexes in *V. natriegens* by introducing the structural operons into vectors, which did not code for additional assembly factors. In case of the NQR, a functional protein complex could be produced without co-expression of *apbE* or *nqrM*. ApbE is a flavin transferase catalyzing the covalent attachment of FMN to NqrB and NqrC in *V. cholerae* (Bertsova et al., 2013). It is necessary for NQR maturation, since the FMNs are required for the transfer of the electrons through the NQR complex. NqrM was predicted to deliver Fe to subunits NqrD and NqrE (Kostyrko et al., 2015). Subunits NqrD and NqrE harbor an iron cluster located in the midst of the membrane part of NQR which is proposed to participate in intramolecular electron transfer (Steuber et al., 2014b). *V. natriegens* was able to produce active NQR, probably because it harbors an endogenous NQR. As in *V. cholerae*, *apbE* and *nqrM* are located immediately downstream of the *nqr* operon in *V. natriegens*. The genes *nqrM* (accession number UNIPROT: A0A1B1EEC2) and *apbE* (accession number UNIPROT: A0A1B1EEE5) from *V. natriegens* are homologous to *nqrM* (accession number UNIPROT: Q9KPS4) and *apbE* (accession number UNIPROT: A5F5Y3) in *V. cholerae*. Both organisms contain two chromosomes. The *apbE*, *nqrM* and the *nqr* genes are located on the larger chromosome (Supplementary Figure S1). Our study shows that ApbE and probably, NqrM, from *V. natriegens* function in maturation of the *V. cholerae* NQR.

Furthermore, we could detect the holo NQR by NBT staining in a BN PAGE. Here a signal at around 185 kDa became visible in the solubilisates (Figure 5, boxes 1, 2) and all NQR subunits could be identified by mass spectrometry. Also at a lower molecular weight level, two signals became visible in each solubilisate sample, indicating two sub-complexes (Figure 5, boxes 3–6). Mass spectrometry identified NqrA and NqrC in the upper protein bands (Figure 5, boxes 3, 4) and NqrA, NqrC, and NqrF (Figure 5, box 6) or NqrA and NqrF (Figure 5, box 5) in the lower ones. We suggest that the upper protein band comprises an Nqr sub-complex of NqrABCF. The formation of such a sub-complex was observed before during purification of the NQR (Tao et al., 2008). It is possible, that NqrF and NqrB, which were not identified in these samples, were washed out before mass spectrometry. When the BN PAGE gel is stained with NBT and not fixed with TCA (trichloroacetic acid) we observed that the proteins are washed out over time and only the purple precipitate remains in the gel matrix. Therefore, we recommend to prepare two BN PAGEs in parallel: One is stained with NBT and the other one is treated with 20% TCA for 30 min and afterward with Coomassie. By comparison of the two gels, the corresponding protein bands are recovered from the TCA-fixed gel for mass spectrometry. The observed depletion of proteins from BN PAGE also offers a rationale for the fact that the upper band stained with NBT apparently obtained only NqrA and NqrC, but not NqrF which represents the only NADH oxidizing subunit in the NQR complex.

While NQR is a primary Na^+^ pump, Mrp represents a secondary transporter catalyzing cation/H^+^ antiport. Like NQR, it is a membrane-bound protein complex composed of six subunits (Swartz et al., 2005; Dzioba-Winogrodzki et al., 2009; Morino et al., 2014, 2017). Functional expression of the Mrp was confirmed by *in vivo* growth experiment demonstrating the rescue of cell growth despite high (toxic) amounts of Li^+^. There was an optimum of expression after 2 h of incubation with 0.02% L-arabinose, followed by subsequent decrease of expression levels. This could reflect increased proteolysis of Mrp, or a decrease in expression levels. We favor the latter explanation because we observed large variations in the capacity of *V. natriegens* transformed with pVc-Mrp to survive high [Li^+^]. In all experiments, pVc-Mrp clearly conferred a growth advantage compared to the empty vector, but the final cell yields observed with pVc-Mrp varied between OD_600_ = 1 at 300 mM LiCl and OD_600_ = 0.5 at 400 mM LiCl. These variations most likely are caused by L-arabinose isomerase (accession number UNIPROT: A0A1B1EI15), an enzyme allowing degradation of the inducer L-arabinose by *V. natriegens*.

In summary, *V. natriegens* is an attractive expression host because of its very high growth rate, its non-pathogenicity, and because it allows scalable expressions of large membrane proteins. Once the metabolism of L-arabinose is blocked by future genetic engineering (Guzman et al., 1995; Baba et al., 2006) *V. natriegens* could become an alternative to *E. coli* as expression host, especially for the overproduction of proteins from pathogenic bacteria.

## Materials and Methods

### Bacterial Strains and Plasmids

*Vibrio natriegens* ATCC 14048 was transformed with the plasmids pBAD-TOPO (Invitrogen pBAD-TOPO^®^ TA), pVc-Mrp (Dzioba-Winogrodzki et al., 2009) or pNqrST (this work) according to the protocol of Weinstock et al. (2016). *E. coli* Top 10 (Invitrogen) was used for the cloning of the pNqrST plasmid.

### Construction and Transformation of Vector pNqrST

The plasmid was constructed for the expression of the NQR from *V. cholerae* carrying a polyhistidine tag and a Strep-Tag at the N-terminus of subunit NqrA. The plasmid is based on the pNQR1 plasmid (Tao et al., 2008) containing the whole *nqr* operon, which consists of the six genes *nqrABCDEF* and a sequence encoding for six histidine residues (His_6_-Tag) fused to the 5′ end of the *nqrA* gene. For the pNqrST construct, the fragment coding for the His_6_-Tag in pNQR1 and a part of the upstream *araB* region were cut out from pNQR1 with the restriction enzymes SgrAI and NdeI. The linearized and truncated pNQR1 plasmid was then ligated with a synthetic SgrAI – NdeI DNA fragment (Eurofins Genomics). This fragment comprises the lost *araB* region, a His_6_-Tag and two streptavidin polypeptides (Supplementary Figure S2). This results in NQR complex with subunit NqrA carrying, at the N-terminus, a His_6_-Tag followed by two consecutive Strep-Tags. Note that pNqrST also confers a protease restriction site (preScission site) for the HRV 3C protease between the histidine residues and the Strep-Tag. Correct cloning was confirmed by sequencing (Eurofins Genomics).

Chemo-competent *E. coli* Top 10 cells were prepared following the protocol of (Inoue et al., 1990). For transformation, an aliquot of 100 μl competent *E. coli* cells was thawed on ice and mixed with 250 ng pNqrST. After incubation on ice for 30 min, cells were heat shocked at 42°C for 30 s and cooled for 5 min on ice. Then 900 μl SOC medium (20 g/l tryptone, 5 g/l yeast extract, 10 mM NaCl, 2.5 mM KCl, 10 mM MgCl_2_, 10 mM MgSO_4_, and 20 mM glucose, pH adjusted to 7.0 with NaOH) was added. After incubation at 37°C for 1 h the cell suspension was centrifuged for 1 min with 11000*g* and 900 μl of the supernatant were discarded. The cell pellet was resuspended in the remaining 100 μl SOC medium and cells were distributed on LB agar plates supplemented with 100 μg/ml ampicillin. Positive clones were identified after plasmid preparation (Miniprep Kit II, peqlab) followed by restriction analyses.

### Growth of *V. natriegens*

Cells were cultivated in LBS medium (10 g/l tryptone, 5 g/l yeast extract, 375 mM NaCl, 4.2 mM KCl, and 23.1 mM MgCl_2_). To obtain a preculture 10 ml LBS medium with 100 μg/ml ampicillin was inoculated with a single colony and cultivated at 30°C on a rotary shaker at 180 rpm overnight. With this preculture 1 L medium was inoculated (1:100) and incubated at 37°C with moderate aeration. At OD_600_ ∼ 0.8 L-arabinose was added to a final concentration of 0.02% to induce the protein expression. *V. natriegens* was stored at -80°C as 15% glycerol stock.

### Membrane Isolation

Cells were harvested after 1–2 h of incubation with L-arabinose by centrifugation at 6000*g* for 30 min. Pellets were resuspended in cell lysis buffer (500 mM KCl, 20 mM potassium phosphate buffer pH 8) at a concentration of 0.2 g/ml and supplemented with protease inhibitor (cOmplete ULTRA Tablets, Roche Diagnostics), 1 mM DTT, 0.5 mM MgCl_2_ and a trace of DNase I. The cells were disrupted in a continuous cell lysis system at 20 kPsi (Emulsiflex C3, Avestin). The suspension was centrifuged with 27000*g* for 30 min to remove the unbroken cells and cell debris. The supernatant was centrifuged with 250000*g* for 1 h to collect the membranes. The pellet was resuspended in buffer A [50 mM potassium phosphate buffer pH 7.5, 5% glycerol (w/v), 300 mM KCl] followed by another centrifugation step in buffer A to wash the membranes. The membranes were resuspended in 5 ml buffer A and frozen in liquid N_2_.

### *In gel* Fluorography

Fluorescence of covalently bound flavins in NqrB and NqrC was detected using the ImageQuant LAS 4000 imager (λ_excitation_ = 460, emission filter = Y515 CyTM2). As a positive control the purified NqrC’ subunit was used. This protein is a truncated variant of the NqrC subunit of the *V. cholerae* NQR comprising the covalently attached FMN but lacking the N-terminal transmembrane helix (Vohl et al., 2014). The molecular mass of NqrC’ is 25.38 kDa.

### PAGE and Activity Staining

Denaturing polyacrylamide gel electrophoresis (SDS-PAGE) was performed with a 12% polyacrylamide gel (Schägger and von Jagow, 1987). Protein and membrane suspensions were diluted in 5× SDS sample buffer [500 mM DTT, 1 M Tris-HCl pH 6.8, 5% (w/v) SDS, 28.8% glycerol, bromophenol blue]. Cell pellets were resuspended in 50 μl 1× SDS sample buffer for cell disruption.

For the Blue native PAGE, SERVA*Gel*^TM^ Native Gels (SERVA) with a gradient from 4 to 16% acrylamide were used. The electrophoresis was performed following the instructions of the manufacturer, modified as follows. First, the gel was run with 30 V for ∼3 h. Then the voltage was increased to 100 V for ∼1.5 h. When 1/3–1/2 of the electrophoresis was completed the blue 1× cathode buffer [50 mM Tricine, 15 mM Bis-Tris pH 7.0, 0.002% (w/v) Coomassie Blue] was changed to 1× cathode buffer (50 mM Tricine, 15 mM Bis-Tris pH 7.0) without Coomassie. Anode buffer remained unchanged (50 mM Bis-Tris pH 7.0). Afterward, the voltage was increased again to 200 V until the blue front reached the bottom of the gel. Activity staining to detect NADH oxidizing proteins was conducted according to the protocol of Yan et al. (2007). Photographs of the gel were taken before activity staining and 30 min or 20 h after incubation in staining solution [50 mM potassium phosphate buffer pH 7.0, 0.2 mg/mL Nitro blue tetrazolium chloride (NBT), 0.1 mg/mL Na_2_NADH]. The purified His-tagged NQR complex (Tao et al., 2008) served as positive control.

### Mass Spectrometry

Nano-LC-ESI-MS/MS experiments were performed on an EASY-nLC 1200 system (Thermo Fisher Scientific, Germany) coupled to a LTQ-Orbitrap XL hybrid mass spectrometer (Thermo Fisher Scientific, Germany) using an EASY-Spray nanoelectrospray ion source (Thermo Fisher Scientific, Germany). Tryptic peptides were directly injected to an EASY-Spray analytical column (PepMap RSLC C18, 2 μm 100 Å 50 μm × 250 mm column, Thermo Fisher Scientific, Germany) operated at constant temperature of 40°C. Gradient elution was performed at a flow rate of 250 nl/min using a 30 min gradient from 2 to 40% solvent B in 30 min, from 40 to 75% in 5 min and final from 75 to 95% in 10 min or rather a 60 min gradient from 2 to 45% in 60 min, in 5 min to 95%. Solvents used were 0.1% formic acid (solvent A) and 80% acetonitrile/0.1% formic acid (end concentration) (solvent B). The LTQ-Orbitrap was operated under the control of Xcalibur software (version 2.1, Thermo Fisher Scientific Inc., United States). Survey spectra (m/z = 200–2000) were detected in the Orbitrap at a resolution of 60,000 at m/z = 400. Data dependent tandem mass spectra were generated for the seven most abundant peptide precursors in the linear ion trap. For all measurements using the Orbitrap detector, internal calibration was performed using lock-mass ions from ambient air as described in Olsen et al. (2005). The data were analyzed by Mascot 2.6 (Matrix Science, United Kingdom), which was used as search engine for protein identification. Spectra were searched against the *V. natriegens* subset of the NCBI protein sequence database downloaded as FASTA-formatted sequences (https://www.ncbi.nlm.nih.gov/, update from December 2016/May 2017). Search parameters specified trypsin as cleaving enzyme, allowing three missed cleavages, a 5 ppm mass 32 tolerance for peptide precursors and 0.6 Da tolerance for fragment ions. Methionine oxidation was allowed as variable modification and carbamidomethylation of cysteine residues was set as fixed modification. The Mascot results were transferred to Scaffold^TM^ Software 4.6.1 (Proteome Software, United States).

### Immune Detection

After separation, proteins were transferred to a nitrocellulose membrane by western blotting (Mini Trans-Blot^®^ Cell system from Bio-Rad). The membrane was incubated for 1 h in blocking solution [137 mM NaCl, 2.7 mM KCl, 1.8 mM KH_2_PO_4_, 10 mM Na_2_HPO_4_, 0.05% Tween, and 5% (w/v) skimmed milk powder]. After two washing steps with PBST (137 mM NaCl, 2.7 mM KCl, 1.8 mM KH_2_PO_4_, 10 mM Na_2_HPO_4_, and 0.05% Tween) for 5 min the nitrocellulose membrane was incubated for 1 h with antibodies against V5-MrpG, or Strep-NqrA, which were diluted 1:5000 in blocking solution. In both cases HRP-conjugated antibodies were used (V5 Tag Monoclonal Antibody E10/V4RR HRP, Thermo Fisher Scientific; Strep-Tactin^®^ conjugated to horseradish peroxidase, iba). After two washing steps with PBST, bound antibodies were detected using chemiluminescence Clarity^TM^ Western ECl Blotting Substrates (Bio-Rad). Exposure (2 min) and detection were performed with the Image Quant LAS 400 (GE Healthcare). For a chromogenic immunodetection, the membrane was incubated with 20 ml PBST, 200 μl 3% 4-Chloro-1-naphthol, and 20 μl 30% H_2_O_2_. As positive control for the immunostaining of Strep-NqrA, the holo NQR-ST complex expressed in *V. cholerae* from the pNqrST plasmid was used. The Strep-His-tagged NQR complex was purified by Nickel affinity chromatography as described for His-tagged NQR complex (Tao et al., 2008).

### Enzyme Kinetics

NADH oxidation assays were carried out under stirring in a quartz cuvette (1 cm diameter) in a total volume of 1 ml at 25°C using a Diode-Array Spectrophotometer. NADH oxidation was followed at 340 nm (𝜀_NADH_ = 6.22 mM^-1^) (Juárez et al., 2009). To avoid precipitation of AgCl, NQR activity in the presence of Ag^+^ was measured in reaction buffer containing 20 mM Tris H_2_SO_4_ pH 8, 100 mM Na_2_SO_4_, 100 μM ubiquinone, and 100 μM Na_2_NADH. A total of 20 μg membranes were incubated with varying amounts of AgNO_3_ for 5 min at RT. Membrane aliquots (20 μg protein) were added to reaction buffer and the oxidation of NADH was followed.

### *In vivo* Function of Mrp Monitored in Growth Experiments

*Vibrio natriegens* transformed with pVc-Mrp or with pBAD-TOPO was grown in LBS medium supplemented with 50 mM Tris HCl pH 7.0, 0.1% L-arabinose, 100 μg/ml ampicillin and varying amounts of NaCl, KCl, or LiCl as indicated. For end point measurements, 14 ml sterile glass test tubes were used. Here, 10 ml fresh medium was inoculated with 50 μl cells from arabinose-induced culture (standardized to an OD_600_ = 1). The starting OD_600_ in all cases was 0.05. After 20 h of incubation at 30 °C and moderate aeration, the optical density was measured at 600 nm (WPA biowave CO8000 cell density meter). To record growth curves, 96-well plates (Thermo Fisher Scientific) were used. A total of 200 μl medium inoculated with induced cells was transferred into the plates and the growth was followed using a plate reader (Tecan infinite F200 Pro) measuring the absorbance at 600 nm every 13 min for 24 h. The plate reader allowed shaking (141 rpm) of the plate while incubation at a constant temperature of 37 °C. To standard optical densities determined with the cell density meter and with the plate reader, we introduced a multiplication factor of 0.58 for the latter to account for the difference in optical path length (Toulouse et al., 2017).

## Author Contributions

LS designed the study, performed the experiments, analyzed the data, and drafted the manuscript. VM took part in designing the study. BC constructed the plasmid pNqrST. JP performed the mass spectrometry analyses. BB provided *Vibrio natriegens* and reviewed the manuscript. PD provided the plasmids pVc-Mrp and pBAD-TOPO and reviewed the manuscript. GF took part in designing the study and reviewed the manuscript. JS designed the study and wrote the manuscript.

## Conflict of Interest Statement

The authors declare that the research was conducted in the absence of any commercial or financial relationships that could be construed as a potential conflict of interest.

## Abbreviations

NQR: Na^+^-translocating NADH:quinone oxidoreductase
Mrp: multiple resistance and pH related antiporter
