# Supplementary material for: Vibrio natriegens as Host for Expression of Multisubunit Membrane Protein Complexes
Source: Front Microbiol. 2018 Oct 25;9:2537. doi: 10.3389/fmicb.2018.02537 (PMC6209661; doi:10.3389/fmicb.2018.02537)
Supplement: Supplementary file 1 [file Data_Sheet_1.docx]

Supplementary Material

*Vibrio natriegens* as host for expression of multisubunit membrane protein complexes

Lena Schleicher, Valentin Muras, Björn Claussen, Jens Pfannstiel, Bastian Blombach, Pavel Dibrov, Günter Fritz, Julia Steuber^1^*

*** Correspondence: Julia Steuber, julia.steuber@uni-hohenheim.de**

# Supplementary Figures


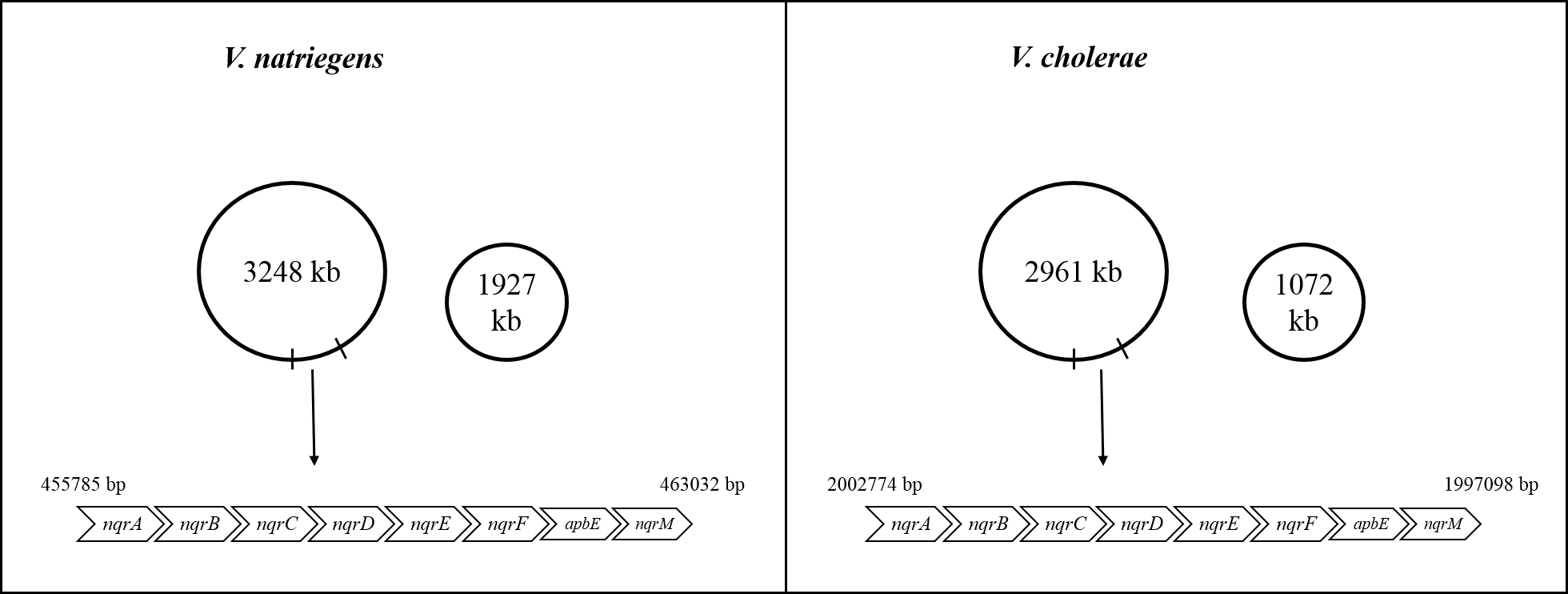


Supplementary Figure S1. Localization of *nqr* genes and the accessory *apbE* and *nqrM* genes on the chromosomes of *V. natriegens* and *V. cholerae*. The *nqr, apbE* and *nqrM* genes are encoded directly behind each other and are located on the larger chromosome of each organism. In both strains there is only one copy of the respective gene. Note that when these sequences are blasted through the NCBI database, two results are displayed for both strains. In the case of *V. cholerae* one sequence is annotated on the first and another on the second chromosome, representing the same sequence obtained from two different sequencing projects. In the case of *V. natriegens,* one sequence is annotated on the plus and one on the minus strand from the larger chromosome, again resulting from two different sequencing projects.


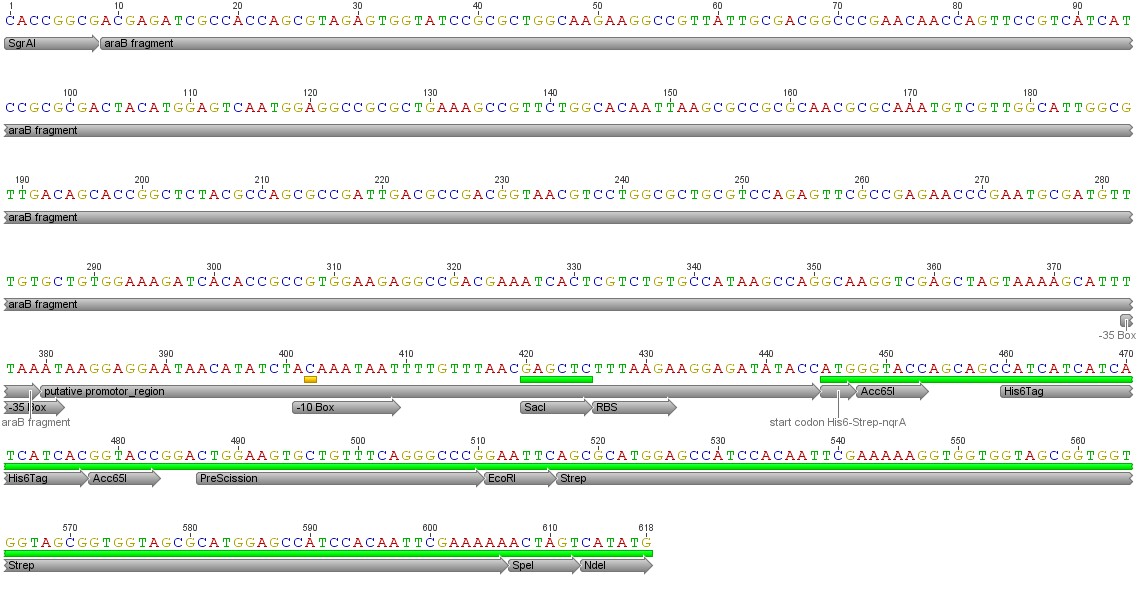


Supplementary Figure S2. Sequence of the His_6_-Strep-Tag region of pNqrST. The His_6_-Strep-Tag upstream of the *nqrA* start codon region comprises a His_6_-Tag, a protease preScission site and the sequence of two streptavidin polypeptides. The NdeI site includes the intrinsic start codon of *nqrA* (red box).

# Supplementary Tables

Supplementary Table S1. Peptide sequences of the different Nqr subunits identified by mass spectrometry. NqrB and NqrC subunits were found by in gel fluorography. The NQR holo-complex was identified by BN PAGE and NBT activity stain, as well as smaller sub-complexes.

| Sequence of identified peptides |  |  |  | NqrA |
| --- | --- | --- | --- | --- |
| NADH dehydrogenases in BN PAGE | *V. natriegens* pBAD-TOPO | Holo-complex  Box 2 |  | KAQVLFEDK  FTAPAAGK  VPAIESSTK  VISLAGPVVNNPR  ELFGWAMPGK  SMVPIGNYER  YEYGQLLR |
|  |  | Sub-complex | Box 4 | VPAIESSTK  VISLAGPVVNNPR  SMVPIGNYER |
|  |  |  | Box 6 | VPAIESSTK  SMVPIGNYER |
|  | *V. natriegens* pNqr-ST | Holo-complex  Box 1 |  | KGLDLPIAGTPSQVINDGK  AQVLFEDKKNPGVKFTAPAAGK  TQLVESGLWTALR  VPAIESSTK  VISLAGPVVNNPR  TTLGASLDDVTDNELMPGEVR  ELFGWAMPGK  SFLGHLFK  SMVPIGNYERVMPLDMEPTLLLR |
|  |  | Sub-complex | Box 3 | VISLAGPVVNNPR |
|  |  |  | Box 5 | AQVLFEDK  VPAIESSTK  SMVPIGNYER |

| Sequence of identified peptides |  |  | NqrB |
| --- | --- | --- | --- |
| Fluorescent proteins in SDS PAGE of membranes | *V. natriegens* pBAD-TOPO |  | FIEDIEHHFEPGGK  EIFGGTGRNFLNPALAGR |
|  | *V. natriegens* pNqr-ST |  | FIEDIEHHFEPGGK  NFLNPALAGR |
| NADH dehydrogenases in BN PAGE | *V. natriegens* pBAD-TOPO | Holo-complex  Box 2 | EIFGGTGRNFLNPALAGR |
|  | *V. natriegens* pNqr-ST | Holo-complex  Box1 | EIFGGTGRNFLNPALAGR |

| Sequence of identified peptides |  |  |  | NqrC |
| --- | --- | --- | --- | --- |
| Fluorescent proteins in SDS PAGE of membranes | *V. natriegens* pBAD-TOPO |  |  | IVEVAGIDASGKKVPELFAQYIEPRLVDFNTGDFVEGNAATYDQRKAAKDPAESIKLSAEDDKAK  RANTGVVYLVK  KLFDENHKPAIK |
|  | *V. natriegens* pNqr-ST |  |  | ASNNDSIKK  ANAVLDKQSKIVEVAGIDASGKKVPELFAQYIEPRLVDFNTGDFVEGNAATYDQRKAAKDPAESIKLSAEDDKAK  RANTGVVYLVK  KLFDENHKPAIK |
| NADH dehydrogenases in BN PAGE | *V. natriegens* pBAD-TOPO | Holo-complex  Box 2 |  | ASNNDSIKK  IVEVAGIDASGK  LSAEDDKAK  ANTGVVYLVK |
|  |  | Sub-complex | Box 4 | AAKDPAESIKLSAEDDKAK  ANTGVVYLVK |
|  |  |  | Box 6 | IVEVAGIDASGK  AAKDPAESIKLSAEDDKAK  ANTGVVYLVK |
|  | *V. natriegens* pNqr-ST | Holo-complex  Box 1 |  | ASNNDSIKK  ANAVLDKQSKIVEVAGIDASGKKVPELFAQYIEPRLVDFNTGDFVEGNAATYDQRKAAKDPAESIKLSAEDDKAK  ANTGVVYLVK  KLFDENHKPAIK |
|  |  | Sub-complex | Box 3 | LSAEDDKAK  ANTGVVYLVK |

| Sequence of identified peptides |  |  | NqrD |
| --- | --- | --- | --- |
| NADH dehydrogenases in BN PAGE | *V. natriegens* pBAD-TOPO | Holo-complex  Box 2 | NHIPNSVR  AYLYDISK |
|  | *V. natriegens* pNqr-ST | Holo-complex  Box1 | AYLYDISK |

| Sequence of identified peptides |  |  | NqrE |
| --- | --- | --- | --- |
| NADH dehydrogenases in BN PAGE | *V. natriegens* pBAD-TOPO | Holo-complex  Box 2 | YSDVPPGLR |

| Sequence of identified peptides |  |  |  | NqrF |
| --- | --- | --- | --- | --- |
| NADH dehydrogenases in BN PAGE | *V. natriegens* pBAD-TOPO | Holo-complex  Box 2 |  | LACQVAMK  SHIFDQLKR  MSFWYGAR |
|  |  | Sub-complex | Box 6 | LACQVAMK  SHIFDQLKR |
|  | *V. natriegens* pNqr-ST | Holo-complex  Box 1 |  | SGGGDILPTELDHITK  LACQVAMKTDMDIELPEEIFGVK  ELKLQIPDGESVPFR  YESKVNEETIR  CTISGPFGEFFAKDTDAEMVFIGGGAGMAPMRSHIFDQLKR  MSFWYGAR  DLGVEDENILLDDFGG |
|  |  | Sub-complex | Box 5 | LACQVAMK  SHIFDQLKR  MSFWYGAR |
